# Real-World Effectiveness and Safety of Immune Checkpoint Inhibitors Combined with Chemotherapy in Taiwanese Patients with Extensive-Stage Small Cell Lung Cancer

**DOI:** 10.3390/curroncol32080472

**Published:** 2025-08-19

**Authors:** Cheng-Yu Chang, Yu-Feng Wei, Shih-Chieh Chang, Chung-Yu Chen

**Affiliations:** 1 Division of Chest Medicine, Department of Internal Medicine, Far Eastern Memorial Hospital, New Taipei City 220216, Taiwan; femh85874@femh.org.tw; 2 Department of Electrical Engineering, Yuan Ze University, Taoyuan City 32003, Taiwan; 3 Department of Internal Medicine, E-Da Cancer Hospital, Kaohsiung 82445, Taiwan; ed102746@edah.org.tw; 4 School of Medicine for International Students, College of Medicine, I-Shou University, Kaohsiung 82445, Taiwan; 5 Division of Chest Medicine, Department of Internal Medicine, National Yang Ming Chiao Tung University Hospital, Yilan 260006, Taiwan; 6 Division of Pulmonary and Critical Care Medicine, Department of Internal Medicine, National Taiwan University Hospital Yunlin Branch, Yunlin County 64002, Taiwan; 7 College of Medicine, National Taiwan University, Taipei 10051, Taiwan

**Keywords:** small cell lung cancer, immunotherapy, chemotherapy, real-world, survival

## Abstract

Purpose: Extensive-stage small cell lung cancer (ES-SCLC) has poor prognosis. While immune checkpoint inhibitors (ICIs) with chemotherapy show survival benefits in trials, real-world data from Asia are scarce. This study evaluates real-world efficacy and safety of chemotherapy with or without ICIs in Taiwanese patients with ES-SCLC and identifies survival predictors. Materials and Methods: A retrospective cohort study analyzed 114 patients with ES-SCLC treated between 2017 and 2023 at four Taiwanese medical centers. Patients received first-line chemotherapy alone (*n* = 68) or with ICIs (atezolizumab, durvalumab, pembrolizumab; *n* = 46). Primary endpoints were overall survival (OS) and progression-free survival (PFS), assessed via Kaplan–Meier methods and Cox regression. Results: Baseline characteristics were comparable, except poorer ECOG performance (≥2) in the chemotherapy group (27% vs. 9%; *p* = 0.021). IO–chemotherapy significantly improved OS (16.1 vs. 9.4 months; HR = 0.32, 95% CI: 0.20–0.52; *p* < 0.001) and PFS (7.8 vs. 5.5 months; HR = 0.40, 95% CI: 0.26–0.63; *p* < 0.001). Multivariate analysis confirmed IO–chemotherapy as an independent positive predictor (OS adjusted HR = 0.25, 95% CI: 0.14–0.44; PFS adjusted HR = 0.37, 95% CI: 0.22–0.61; both *p* < 0.001). Skin rash was more common with IO–chemotherapy (24% vs. 3%; *p* < 0.001). Immune-related adverse events (AEs) correlated with improved survival (median OS: 21.4 months with 1–2 AEs, 16.6 months with 3–4 AEs, 12.5 months without AEs). Conclusion: Immunochemotherapy significantly improves survival in Taiwanese patients with ES-SCLC, with manageable toxicity, supporting ICIs’ incorporation into standard treatment.

## Simple Summary

This real-world retrospective study evaluated the effectiveness and safety of immune checkpoint inhibitors (ICIs) combined with chemotherapy in Taiwanese patients with extensive-stage small cell lung cancer (ES-SCLC). ICIs significantly improved overall and progression-free survival, and the occurrence of immune-related adverse events correlated with favorable survival outcomes. These results advocate for the incorporation of ICIs into standard clinical care for patients with ES-SCLC in Asian populations.

## 1. Introduction

Small cell lung cancer (SCLC) is an aggressive subtype of lung cancer, accounting for approximately 15% of cases, and is closely linked to tobacco exposure [1]. It is classified as limited-stage (LS-SCLC) or extensive-stage (ES-SCLC) according to the AJCC staging [2]. Platinum-based chemotherapy has long been the standard treatment, but despite numerous trials, survival outcomes remain poor, with progression-free survival (PFS) under 6 months and median overall survival (OS) around 10 months [3,4].

Immune checkpoint inhibitors, such as the PD-L1 inhibitor atezolizumab, have introduced new hope. The IMpower133 trial demonstrated improved survival with atezolizumab plus chemotherapy in ES-SCLC, showing a median OS of 12.3 months versus 10.3 months for placebo (HR 0.70), and a significant PFS improvement (HR 0.77) [5]. Similarly, the CASPIAN trial found durvalumab plus chemotherapy improved OS (HR 0.73), with a median OS of 13.0 months compared with 10.3 months for chemotherapy alone, though PFS improvement was not significant [6]. In contrast, the KEYNOTE-604 trial, which evaluated pembrolizumab with chemotherapy, failed to show a significant OS improvement despite a PFS benefit [7]. These studies confirm that PD-L1 inhibitors combined with chemotherapy can improve survival in ES-SCLC, but questions remain regarding patient selection and the role of PD-1 inhibitors.

This study aims to evaluate the real-world effectiveness and safety of immunotherapy in Taiwanese patients with extensive-stage small cell lung cancer (ES-SCLC). It investigates the progression-free survival (PFS) and overall survival (OS) outcomes of combining immune checkpoint inhibitors (such as PD-1 or PD-L1 inhibitors) with chemotherapy, comparing them against traditional treatment approaches. It also assesses the safety profile, focusing on immune-related adverse events including pneumonitis and dermatitis. Subgroup analyses will examine differential treatment responses based on variables such as age, sex, smoking history, molecular markers, and comorbidities. Real-world treatment patterns in Taiwan—such as combination therapy regimens, treatment duration, and discontinuation reasons—will be analyzed for their impact on prognosis. This study also explores long-term survival trends and recurrence patterns post-immunotherapy and compares these findings with global clinical trial data (e.g., IMpower133, CASPIAN) to determine consistency and explore reasons for any observed discrepancies. This comprehensive evaluation will help bridge the gap between clinical trial efficacy and real-world applicability in the Taiwanese population.

## 2. Materials and Methods

### 2.1. Study Design and Data Collection

This retrospective observational study was conducted across four medical centers in Taiwan—National Taiwan University Hospital Yunlin Branch in Yunlin, E-DA Hospital in Kaohsiung, Far Eastern Memorial Hospital in New Taipei City, and National Yang Ming University Hospital in Yilan—over a five-year period from 1 January 2019 to 31 December 2023. Eligible participants were adults (≥18 years) with a pathologically confirmed diagnosis of extensive-stage small cell lung cancer (ES-SCLC) who had received at least one cycle of immune checkpoint inhibitor (ICI) therapy (e.g., PD-1 or PD-L1 inhibitors) combined with chemotherapy as initial treatment. Additional inclusion criteria required complete clinical records, including treatment dates, follow-up data, response assessments, and documentation of adverse events, with at least six months of follow-up or earlier events such as progression or death. Patients with concurrent primary malignancies (unless cured and disease-free for more than five years), those enrolled in other interventional trials during the study period, or individuals lacking sufficient data were excluded. All eligible patients were identified and enrolled based on these criteria. This study received expedited Institutional Review Board (IRB) approval of the National Taiwan University Hospital (IRB No.: 202504087RIND).

Patients were divided into two cohorts based on their initial treatment: those who received first-line immunotherapy combined with chemotherapy, and those who received first-line conventional chemotherapy alone. Demographic information, treatment details, and clinical outcomes were extracted from standardized electronic medical records, including patient demographics (age, sex), medical and surgical history, laboratory findings, imaging studies, pulmonary function test results, and medication usage. Imaging evaluations and treatment response assessments were conducted every 3–4 months after enrollment, and subsequent analysis focused on progression-free survival (PFS) and overall survival (OS).

### 2.2. Adverse Events Classification

Adverse events were monitored throughout the treatment period and were classified and graded according to the Common Terminology Criteria for Adverse Events (CTCAE), version 5.0. Adverse events were categorized according to treatment type and monitored throughout this study. Immunotherapy-related adverse events included dermatologic reactions (such as rash and pruritus), immune-related pneumonitis, hepatitis with elevated liver enzymes and jaundice, various endocrinopathies (including thyroid dysfunction, adrenal insufficiency, and pituitary disorders), immune-mediated colitis, nephritis and renal impairment, cardiotoxicity (including myocarditis and pericarditis), and other less common immune-related events such as pancreatitis and neuritis. Chemotherapy-related adverse events included myelosuppression (manifested as leukopenia, thrombocytopenia, and anemia), nausea and vomiting, alopecia, nephrotoxicity, neurotoxicity (particularly peripheral neuropathy), oral mucositis, and a range of gastrointestinal symptoms such as diarrhea, constipation, and anorexia.

### 2.3. Statistical Analysis

Descriptive statistics summarized baseline characteristics using means and standard deviations for continuous variables and frequencies for categorical variables. Survival outcomes were assessed using the Kaplan–Meier method, and differences between treatment groups were evaluated via the log-rank test. To control for potential confounders such as age, sex, and ECOG performance status, multivariate analysis was performed using the Cox proportional hazards model. Univariate analyses compared continuous variables with Student’s *t*-test (for two groups) or one-way ANOVA (for multiple groups), while categorical variables were analyzed using the chi-square test.

## 3. Results

### 3.1. Participates Enrollment

**Figure 1:**
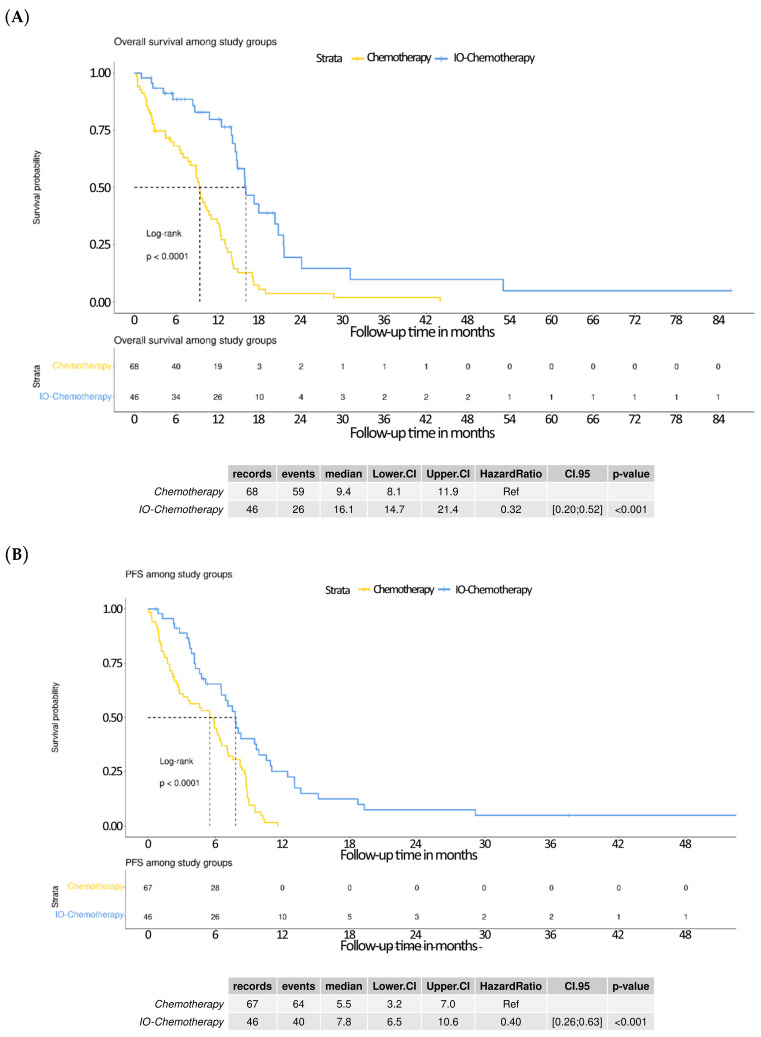
Kaplan–Meier curves comparing survival outcomes between treatment groups. (**A**) Overall survival (OS): patients treated with immunotherapy plus chemotherapy (IO–chemotherapy) demonstrated a significantly longer median OS of 16.1 months (95% CI: 14.7–21.4) compared with 9.4 months (95% CI: 8.1–11.9) in the chemotherapy-only group, with a hazard ratio (HR) of 0.32 (95% CI: 0.20–0.52, *p* < 0.0001, log-rank test). (**B**) Progression-free survival (PFS): similarly, IO–chemotherapy resulted in an improved median PFS of 7.8 months (95% CI: 6.5–10.6) compared with 5.5 months (95% CI: 3.2–7.0) for patients receiving chemotherapy alone (HR 0.40, 95% CI: 0.26–0.63, *p* < 0.0001, log-rank test).

Patients diagnosed with extensive-stage small cell lung cancer (SCLC) from four medical centers in Taiwan—National Yang Ming University Hospital (NYCUH, *n* = 17), National Taiwan University Hospital Yunlin Branch (*n* = 63), Far Eastern Memorial Hospital (FEMH, *n* = 25), and E-DA Hospital (EDA, *n* = 15)—were enrolled, resulting in a total of 120 cases. Following the exclusion of 6 patients—2 without treatment data and 4 lacking survival information—a final cohort of 114 patients was included in this study. These individuals were categorized into two groups based on their first-line treatment: 68 patients received chemotherapy alone, while 46 received a combination of immunotherapy and chemotherapy (IO–chemotherapy). For outcome evaluation, overall survival (OS) was analyzed for all patients, whereas progression-free survival (PFS) and time on treatment (ToT) were assessed in 67 patients from the chemotherapy group and all 46 patients from the IO–chemotherapy group (Figure 1).

### 3.2. Basic Characteristics

Both treatment groups were similar in age distribution and predominantly male, with comparable nutritional status as reflected by BMI. Nearly all patients had a history of smoking. A significantly higher proportion of patients in the IO–chemotherapy group had good performance status (ECOG 0–1) compared with those in the chemotherapy group (91% vs. 73%, *p* = 0.021), while poor performance status (ECOG ≥ 2) was more common in the chemotherapy group. Although the overall metastatic burden did not differ significantly between the groups, the lower rate of brain metastases in the IO–chemotherapy group may reflect selective treatment practices. Laboratory parameters—including WBC, segmented neutrophils, lymphocytes, neutrophil-to-lymphocyte ratio (NLR), liver enzymes (AST, ALT), and serum creatinine—were also comparable between groups, with no statistically significant differences observed (Table 1).

### 3.3. Treatment Response and Adverse Events

The median number of chemotherapy cycles was comparable between groups, with both receiving a median of 5 cycles, though the IO–chemotherapy group had a narrower interquartile range (4–6 vs. 2–6). The IO–chemotherapy group demonstrated a significantly higher partial response rate (64% vs. 33%; *p* = 0.005), while progressive disease was more prevalent in the chemotherapy group (56% vs. 27%), indicating greater treatment efficacy with the addition of immunotherapy (Table 1).

Immune-related skin rash was significantly more frequent in the IO–chemotherapy group (24% vs. 2.9%; *p* < 0.001). Endocrinal disorders and brain metastases were slightly more common in the IO–chemotherapy group, with borderline statistical significance (*p* = 0.081 and *p* = 0.091, respectively). Additionally, the IO–chemotherapy group had a significantly longer median follow-up duration (13 vs. 9 months; *p* = 0.001) (Table 1).

Among the 16 patients with ECOG performance status (PS) 2–3, treatment-related adverse events were generally more frequent than in the overall cohort. Immune-related toxicities included skin rash (6.3%), liver toxicity (6.3%), diarrhea (6.3%), and endocrine disorders (6.3%), while interstitial lung disease was not observed in this subgroup. Acute kidney injury occurred in one patient (6.3%). Hematologic toxicities were common, with neutropenia observed in the majority of patients (mean neutrophil count reduction: 242 × 10^6^/L), anemia present in 43.8%, and thrombocytopenia in 31.3%. Treatment discontinuation due to adverse events occurred in one patient (6.3%), and one treatment-related death (6.3%) was reported.

### 3.4. Treatment Outcomes and Patient Survival

The IO–chemotherapy group demonstrated significantly improved overall survival (OS) compared with the chemotherapy group, as shown in the Kaplan–Meier analysis: median OS was 16.1 months (95% CI: 14.7–21.4) versus 9.4 months (95% CI: 8.1–11.9), with a hazard ratio (HR) of 0.32 (95% CI: 0.20–0.52; *p* < 0.001; Figure 1A). Similarly, the median progression-free survival (PFS) was longer in the IO–chemotherapy group at 7.8 months (95% CI: 6.5–10.6) compared with 5.5 months (95% CI: 3.2–7.0) in the chemotherapy group, with an HR of 0.40 (95% CI: 0.26–0.63; *p* < 0.001; Figure 1B). These results indicate that IO–chemotherapy offers a significant and clinically meaningful advantage in both delaying disease progression and extending survival in patients with extensive-stage small cell lung cancer in a real-world setting.

**Figure 2:**
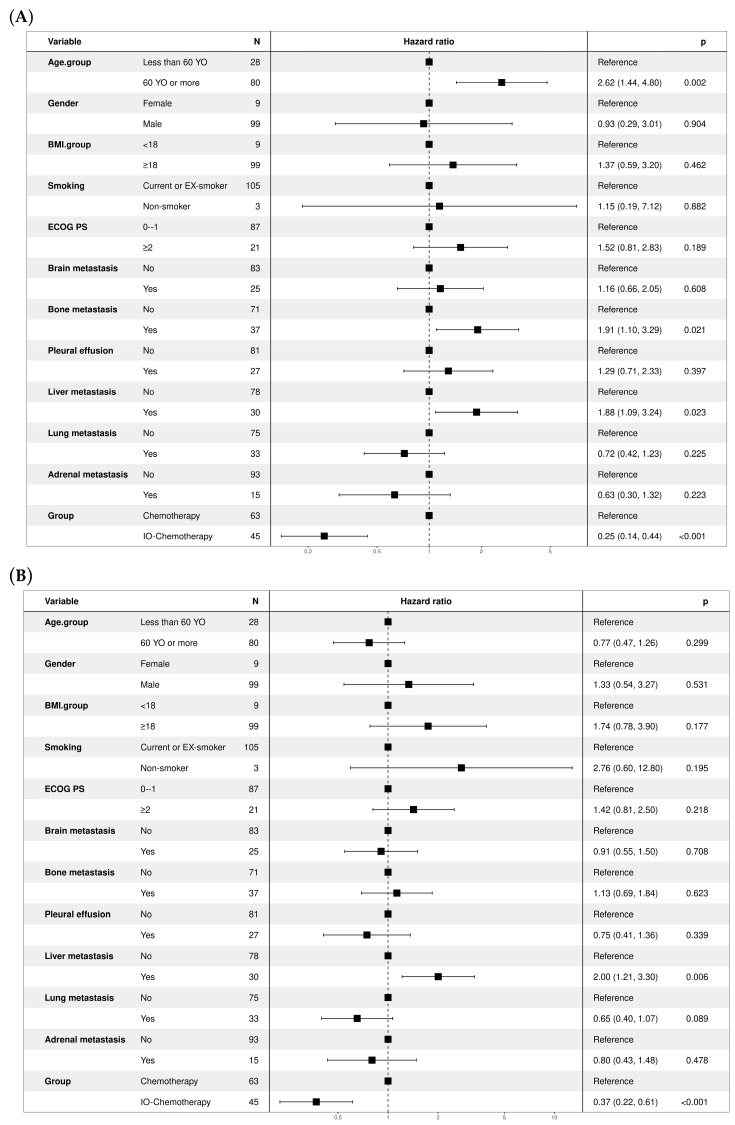
Forest plots for potential predictors of survival outcomes. Multivariate Cox regression analysis evaluating various clinical factors as predictors of survival in patients with extensive-stage small cell lung cancer. (**A**) Overall survival (OS): IO–chemotherapy was associated with significantly improved survival (HR 0.25; 95% CI: 0.14–0.44; *p* < 0.001). Age ≥ 60 years (HR 2.62; *p* = 0.002), bone metastasis (HR 1.91; *p* = 0.021), and liver metastasis (HR 1.88; *p* = 0.023) were significant negative predictors. (**B**) Progression-free survival (PFS): IO–chemotherapy also significantly improved PFS (HR 0.37; 95% CI: 0.22–0.61; *p* < 0.001). Liver metastasis was associated with worse PFS (HR 2.00; *p* = 0.006).

Multivariate Cox regression analysis identified IO–chemotherapy as a strong independent predictor of improved overall survival compared with chemotherapy alone. The adjusted hazard ratio (HR) for IO–chemotherapy was 0.25 (95% CI: 0.14–0.44, *p* < 0.001) (Figure 2A). Other significant predictors included age ≥ 60 years (HR = 2.62, 95% CI: 1.44–4.80, *p* = 0.002), bone metastasis (HR = 1.91, 95% CI: 1.10–3.29, *p* = 0.021), and liver metastasis (HR = 1.88, 95% CI: 1.09–3.24, *p* = 0.023), all associated with poorer prognosis. IO–chemotherapy was also significantly associated with better progression-free survival, with an adjusted HR of 0.37 (95% CI: 0.22–0.61, *p* < 0.001) (Figure 2B). Among clinical variables, liver metastasis emerged as a significant negative prognostic factor (HR = 2.00, 95% CI: 1.21–3.30, *p* = 0.006), while other factors such as age, ECOG performance status, and brain or lung metastases did not reach statistical significance in the multivariate model.

### 3.5. Subgroup Analysis Comparing Atezolizumab and Durvalumab

**Figure 3:**
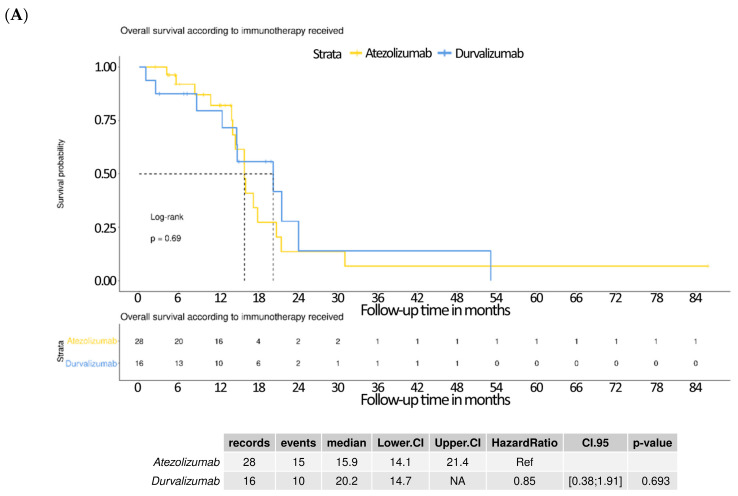

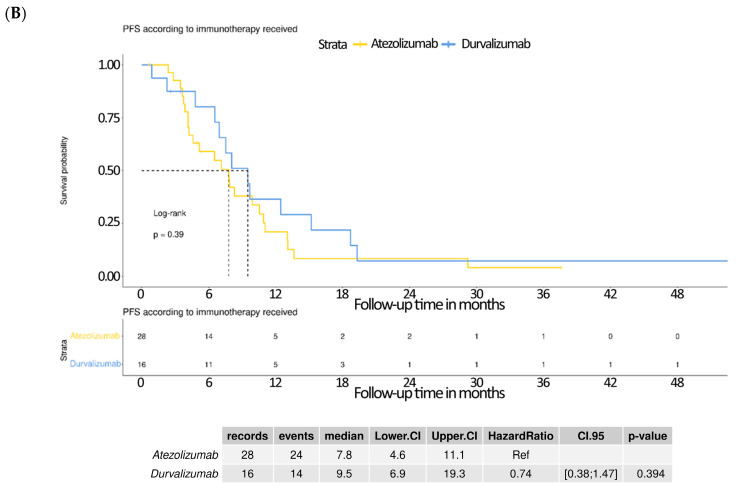
Kaplan–Meier survival curves comparing outcomes according to type of immunotherapy received. (**A**) Overall survival (OS): there was no significant difference in OS between patients treated with atezolizumab and those treated with durvalumab (median OS: atezolizumab 15.9 months [95% CI: 14.1–21.4], durvalumab 20.2 months [95% CI: 14.7–NA]; hazard ratio [HR] 0.85 [95% CI: 0.38–1.91]; *p* = 0.693). (**B**) Progression-free survival (PFS): similarly, no significant difference was observed in PFS between the two immunotherapies (median PFS: atezolizumab 7.8 months [95% CI: 4.6–11.1], durvalumab 9.5 months [95% CI: 6.9–19.3]; HR 0.74 [95% CI: 0.38–1.47]; *p* = 0.394).

The Kaplan–Meier analysis of overall survival showed no significant difference between patients receiving atezolizumab and those receiving durvalumab. The median OS was 15.9 months (95% CI: 14.1–21.4) for the atezolizumab group and 20.2 months (95% CI: 14.7–NA) for the durvalumab group. The hazard ratio (HR) for durvalumab relative to atezolizumab was 0.85 (95% CI: 0.38–1.91; *p* = 0.693), indicating no statistically meaningful survival advantage between the two agents (Figure 3A). No significant difference was also observed in progression-free survival. The median PFS was 7.8 months (95% CI: 4.6–11.1) for the atezolizumab group and 9.5 months (95% CI: 6.9–19.3) for the durvalumab group. The hazard ratio was 0.74 (95% CI: 0.38–1.47; *p* = 0.394), again showing no statistically significant difference (Figure 3B).

### 3.6. Survival Outcomes Stratified by Adverse Events in the IO–Chemotherapy Group

**Figure 4:**
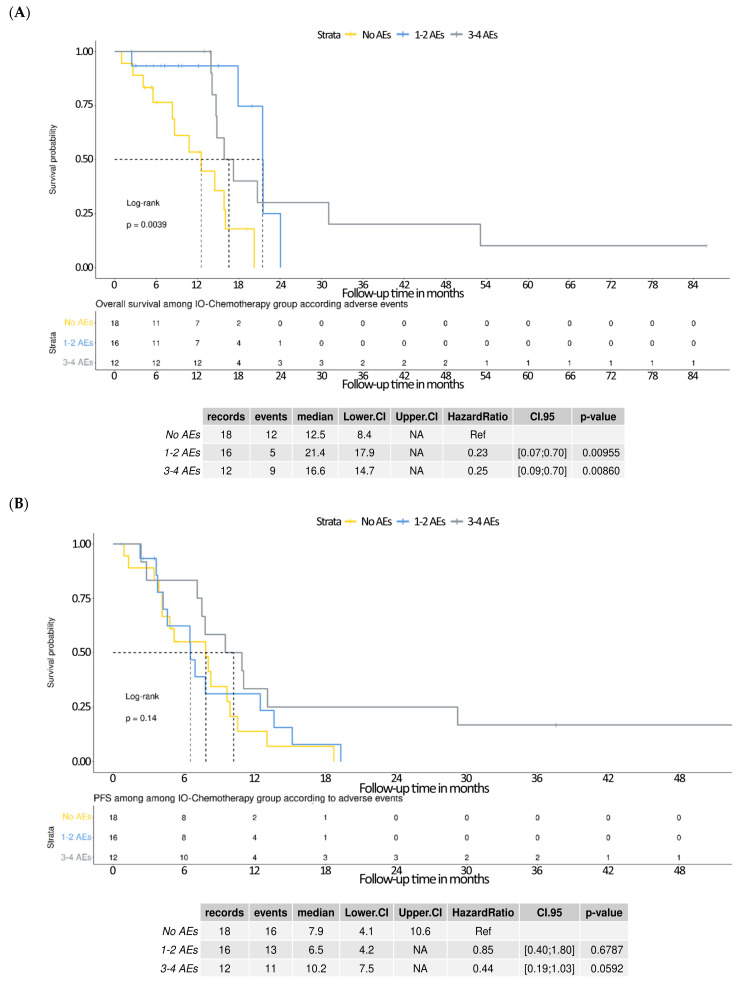
Kaplan–Meier survival analysis of the IO–chemotherapy group stratified by the number of immune-related adverse events (irAEs). (**A**) Overall survival (OS) curves demonstrate a significant difference between groups, with patients experiencing 1–2 or ≥3 irAEs showing prolonged OS compared with those without irAEs (log-rank *p* = 0.0029). Median OS was 21.4 months for 1–2 AEs, 16.6 months for ≥3 AEs, and 12.5 months for no AEs. Multivariable Cox regression indicated significantly lower hazard ratios for both 1–2 AEs (HR = 0.23, 95% CI: 0.07–0.70, *p* = 0.00955) and ≥3 AEs (HR = 0.25, 95% CI: 0.09–0.70, *p* = 0.00660), using the no AE group as reference. (**B**) Progression-free survival (PFS) curves show a trend toward improved outcomes in patients with ≥3 AEs, though the difference was not statistically significant (log-rank *p* = 0.14). Median PFS was 6.5 months in the 1–2 AEs group, 8.4 months in the ≥3 AEs group, and 7.9 months in the no AEs group. Hazard ratios for PFS were 0.85 (95% CI: 0.40–1.80, *p* = 0.6787) for 1–2 AEs and 0.41 (95% CI: 0.19–1.00, *p* = 0.0592) for ≥3 AEs, compared with the reference group.

Kaplan–Meier analyses were performed to evaluate overall survival (OS) and progression-free survival (PFS) among patients receiving IO–chemotherapy, stratified by the number of adverse events (AEs). The OS outcomes differed significantly across groups (log-rank *p* = 0.0039). Patients who experienced 1–2 AEs had a median OS of 21.4 months, with a hazard ratio (HR) of 0.23 (95% CI: 0.07–0.70, *p* = 0.0096) compared with those with no AEs (median OS: 12.5 months). Similarly, patients with 3–4 AEs demonstrated a median OS of 16.6 months and an HR of 0.25 (95% CI: 0.09–0.70, *p* = 0.0086) (Figure 4A). These results suggest that the occurrence of immune-related AEs may be associated with favorable survival outcomes. In contrast, the Kaplan–Meier analysis of PFS showed no statistically significant difference across AE subgroups (log-rank *p* = 0.14), though a favorable trend was observed. Median PFS was 7.9 months in the no AE group, 6.5 months in the 1–2 AEs group (HR = 0.85; 95% CI: 0.40–1.80; *p* = 0.6787), and 10.2 months in the 3–4 AEs group (HR = 0.44; 95% CI: 0.19–1.03; *p* = 0.0592) (Figure 4B). Although not statistically significant, these findings suggest a potential association between higher AE burden and improved PFS. In contrast, chemotherapy-related AEs alone did not appear to influence survival outcomes.

## 4. Discussion

This study evaluated the real-world effectiveness and safety of immune checkpoint inhibitor (ICI)-based therapy combined with chemotherapy (IO–chemotherapy) compared with chemotherapy alone in Taiwanese patients with extensive-stage small cell lung cancer (ES-SCLC). Consistent with previous landmark trials such as IMpower133 and CASPIAN, the current analysis demonstrated significant clinical benefits of IO–chemotherapy in both progression-free survival (PFS) and overall survival (OS). Patients receiving IO–chemotherapy achieved a median OS of 16.1 months compared with 9.4 months with chemotherapy alone, representing a substantial reduction in mortality risk. Similarly, PFS improved markedly from 5.5 months in the chemotherapy group to 7.8 months in the IO–chemotherapy group. These outcomes highlight immunotherapy’s robust efficacy in a real-world Taiwanese patient population, confirming and even surpassing findings from global trials.

Treatment responses correlated closely with survival improvements, as demonstrated by significantly higher rates of partial response and lower disease progression in the IO–chemotherapy group. Crucially, subgroup analyses verified IO–chemotherapy as an independent favorable prognostic factor, unaffected by patient demographic variables or patterns of metastatic spread [8,9,10]. Evidence suggests that the effectiveness of chemoimmunotherapy extends broadly across various demographic groups, including age and gender, and remains consistent regardless of metastatic sites, such as liver, brain, or bone involvement [11]. Although comparisons between atezolizumab and durvalumab revealed no significant differences, both ICIs delivered comparable survival outcomes, highlighting their therapeutic equivalence [12].

In our analysis of the relationship between adverse events and survival, we observed that patients experiencing 1–2 immune-related adverse events (irAEs) had the most pronounced survival advantage, whereas those with ≥3 AEs showed numerically lower—but not statistically significant—overall survival compared with the 1–2 AE group. This pattern suggests that moderate irAEs may serve as markers of robust immune activation, correlating with improved therapeutic efficacy, whereas multiple or severe irAEs could reflect more extensive immune system activation leading to clinically significant toxicity. Such toxicity may necessitate treatment interruptions, dose modifications, or premature discontinuation, potentially diminishing the therapeutic benefit. This interpretation aligns with prior reports in both NSCLC and SCLC populations [13], where the prognostic value of irAEs was most apparent in cases of mild to moderate severity, while excessive toxicity was associated with reduced treatment exposure. Regarding safety, immune-related skin rash was significantly more frequent in patients receiving IO–chemotherapy, consistent with known immune-related adverse events [13]. Other toxicities, including endocrinal disorders and interstitial lung disease, were infrequent and generally mild, confirming acceptable tolerability profiles [14]. While increased follow-up duration in the IO–chemotherapy group may have influenced adverse event detection, treatment discontinuations due to toxicity remained rare [14,15]. Despite the increased baseline vulnerability of patients with PS 2–3, most toxicities were manageable with standard supportive care measures, and the majority of patients completed the planned treatment regimen.

In this study involving patients with extensive-stage small cell lung cancer (ES-SCLC) treated with a combination of immune-oncology (IO) therapy and chemotherapy, a significant association was identified between the occurrence of immune-related adverse events (irAEs) and improved overall survival (OS). Patients who experienced 1–4 irAEs demonstrated notably longer median OS compared with those without irAEs, supporting the notion that irAEs may reflect enhanced immune activation and therapeutic efficacy. Although the trend toward longer progression-free survival (PFS) in patients with multiple irAEs did not achieve statistical significance, the survival patterns suggest a potential link between irAEs and sustained antitumor responses. These findings are in line with previous observations in non-small cell lung cancer (NSCLC) populations [16,17], and further reinforced by studies in SCLC showing that the presence of irAEs is predictive of better clinical outcomes. Notably, patients who developed multi-system irAEs exhibited superior survival compared with those with single-system or no irAEs [18]. In contrast, adverse events related solely to chemotherapy did not correlate with survival outcomes in this cohort, emphasizing the distinct prognostic relevance of irAEs and underscoring the immunologic mechanisms underpinning the clinical benefit of IO-based therapies in SCLC.

When comparing our real-world findings with pivotal trials such as IMpower133, CASPIAN, and KEYNOTE-604, several differences should be considered. First, our cohort consisted entirely of Asian patients treated in routine clinical practice, including individuals with ECOG performance status ≥ 2 and significant comorbidities who are typically excluded from phase III trials. Second, IO–chemotherapy regimens in our study were administered under real-world conditions, with variability in treatment schedules, AE monitoring, and supportive care, rather than the protocol-driven uniformity of clinical trials. Third, follow-up intervals for imaging and AE assessments reflected standard care rather than intensive trial protocols, which may have influenced both detection rates and timing of reported events. Despite these differences, our real-world results demonstrated a survival benefit for IO–chemotherapy consistent with those observed in randomized controlled trials, supporting the applicability of this approach to broader less-selected patient populations.

Despite its strengths, including a robust real-world dataset and comprehensive survival analyses, this study has limitations inherent to retrospective designs, such as potential selection bias and incomplete data collection. It is worth noting that irAEs can vary in type and severity, and not all may confer the same prognostic value. The magnitude of benefit and the AE profile showed some variation, likely attributable to differences in baseline patient characteristics, treatment adherence, and healthcare system factors. These distinctions highlight the importance of real-world data in complementing clinical trial evidence and guiding personalized treatment decisions in diverse patient populations. Moreover, as this study is retrospective and observational in nature, the potential for confounding factors such as baseline immune status or tumor burden cannot be excluded. Nonetheless, these findings provide compelling evidence supporting the integration of IO–chemotherapy into standard care for Taiwanese patients with ES-SCLC, emphasizing the need for further prospective studies to validate and expand these results.

## 5. Conclusions

This multicenter real-world study suggests that immune checkpoint inhibitors (ICIs) combined with chemotherapy can provide clinically meaningful improvements in both overall and progression-free survival for Taiwanese patients with extensive-stage small cell lung cancer (ES-SCLC), with an acceptable and manageable safety profile. The occurrence of mild to moderate immune-related adverse events (irAEs) appeared to correlate with improved survival, whereas multiple or more severe irAEs may attenuate benefit, underscoring the need for balanced toxicity management. Notably, patients with a poorer baseline performance status experienced higher rates of adverse events, reinforcing the importance of careful patient selection and close monitoring.

While our findings are consistent with the results of pivotal randomized trials, key differences in patient selection, treatment conditions, and monitoring highlight the necessity for cautious interpretation. Given the retrospective design, modest sample size, and limited follow-up, these results should be viewed as hypothesis-generating. Prospective studies with larger and more diverse Asian populations, extended follow-up, and incorporation of molecular profiling are warranted to confirm these observations and to optimize personalized therapeutic strategies for ES-SCLC. 

## Figures and Tables

**Table 1:** Study characteristics according to treatment group.

Characteristic	Chemotherapy *N* = 68 ^1^	IO–Chemotherapy *N* = 46 ^1^	*p*-Value ^2^
Age	70 (60, 77)	68 (59, 75)	0.5
Unknown	1	0	
Gender			0.8
Male	61 (91%)	41 (89%)	
Female	6 (9.0%)	5 (11%)	
Unknown	1	0	
BMI	22.8 (20.8, 24.4)	23.1 (19.7, 26.4)	0.5
Smoking			>0.9
Current or ex-smoker	66 (97%)	44 (98%)	
Non-smoker	2 (2.9%)	1 (2.2%)	
Unknown	0	1	
ECOG PS			0.021
0–1	47 (73%)	41 (91%)	
≥2	17 (27%)	4 (8.9%)	
Unknown	4	1	
Brain metastasis			0.091
No	48 (71%)	38 (84%)	
Yes	20 (29%)	7 (16%)	
Unknown	0	1	
Bone metastasis			0.5
No	43 (63%)	31 (69%)	
Yes	25 (37%)	14 (31%)	
Unknown	0	1	
Pleural effusion			0.3
No	48 (71%)	36 (80%)	
Yes	20 (29%)	9 (20%)	
Unknown	0	1	
Lung metastasis			0.2
No	45 (66%)	35 (78%)	
Yes	23 (34%)	10 (22%)	
Unknown	0	1	
Liver metastasis			0.8
No	49 (72%)	34 (74%)	
Yes	19 (28%)	12 (26%)	
Adrenal metastasis			0.6
No	60 (88%)	38 (84%)	
Yes	8 (12%)	7 (16%)	
Unknown	0	1	
WBC	8.2 (5.9, 10.3)	7.9 (6.2, 9.1)	0.4
Unknown	0	1	
Seg	73 (63, 83)	72 (65, 79)	0.5
Unknown	0	1	
Lymphocyte	16 (9, 26)	18 (13, 24)	0.3
Unknown	0	1	
NLR	4.7 (2.4, 8.8)	3.9 (2.7, 5.8)	0.3
Unknown	0	1	
AST	26 (19, 43)	24 (18, 31)	0.2
Unknown	6	1	
ALT	24 (14, 39)	20 (15, 25)	0.4
Unknown	1	1	
Total bilirubin	0.54 (0.40, 0.67)	0.47 (0.38, 0.62)	0.2
Unknown	2	2	
Serum creatinine	0.90 (0.70, 1.10)	0.84 (0.70, 1.06)	0.5
Unknown	0	1	
Chemotherapy cycles no.	5.00 (2.00, 6.00)	5.00 (4.00, 6.00)	0.2
Unknown	1	1	
Response			0.005
Partial response	22 (33%)	28 (64%)	
Progressive disease	37 (56%)	12 (27%)	
Stable disease	7 (11%)	4 (9.1%)	
Unknown	2	2	
Brain metastasis progression			0.090
No	48 (73%)	38 (86%)	
Yes	18 (27%)	6 (14%)	
Unknown	2	2	
Skin rash			<0.001
No	66 (97%)	34 (76%)	
Grade 1–2 ADR	2 (2.9%)	11 (24%)	
Unknown	0	1	
Liver toxicity			0.4
No	60 (88%)	43 (96%)	
Grade 1–2 ADR	7 (10%)	2 (4.4%)	
Grade ≥3 ADR	1 (1.5%)	0 (0%)	
Unknown	0	1	
Diarrhea			0.2
No	62 (91%)	37 (82%)	
Grade 1–2 ADR	6 (8.8%)	8 (18%)	
Unknown	0	1	
Interstitial lung disease			0.4
No	68 (100%)	44 (98%)	
Grade 1–2 ADR	0 (0%)	1 (2.2%)	
Unknown	0	1	
Endocrinal disorders			0.081
No	67 (99%)	41 (91%)	
Grade 1-2 ADR	1 (1.5%)	4 (8.9%)	
Unknown	0	1	
AKI			0.4
No	63 (93%)	44 (98%)	
Grade 1–2 ADR	5 (7.4%)	1 (2.2%)	
Unknown	0	1	
Neutropenia			0.5
No	49 (72%)	35 (78%)	
Yes	19 (28%)	10 (22%)	
Unknown	0	1	
Anemia			0.3
No	48 (71%)	36 (80%)	
Yes	20 (29%)	9 (20%)	
Unknown	0	1	
Thrombocytopenia			0.2
No	53 (78%)	30 (67%)	
Yes	15 (22%)	15 (33%)	
Unknown	0	1	
Treatment-related discontinuation			0.5
No	66 (97%)	45 (100%)	
Yes	2 (2.9%)	0 (0%)	
Unknown	0	1	
Treatment-related death			>0.9
No	67 (99%)	45 (100%)	
Yes	1 (1.5%)	0 (0%)	
Unknown	0	1	
Duration of follow-up	9 (3, 12)	13 (6, 17)	0.001

^1^ Median (Q1, Q3); *n* (%); ^2^ Wilcoxon rank sum test; Fisher’s exact test; Pearson’s Chi-squared test.

## Data Availability

Data sharing is not applicable to this article as no datasets were generated or analyzed during the current study beyond those reported within the manuscript.
